# Mismatch negativity generation in subjects at risk for psychosis: source analysis is more sensitive than surface electrodes in risk prediction

**DOI:** 10.3389/fpsyt.2023.1130809

**Published:** 2023-07-19

**Authors:** Tina Aeberli, Mario Müller, Anastasia Theodoridou, Florence Hagenmuller, Erich Seifritz, Susanne Walitza, Wulf Rössler, Wolfram Kawohl, Karsten Heekeren

**Affiliations:** ^1^Department of Psychiatry, Psychotherapy and Psychosomatics, University of Zurich, Zurich, Switzerland; ^2^The Zurich Program for Sustainable Development of Mental Health Services (ZInEP), University of Zurich, Zurich, Switzerland; ^3^Department of Child and Adolescent Psychiatry and Psychotherapy, University of Zurich, Zurich, Switzerland; ^4^Department of Psychiatry and Psychotherapy, Charité University Medicine, Berlin, Germany; ^5^Clienia Schlössli AG, Oetwil am See, Zurich, Switzerland; ^6^University of Nicosia Medical School, Nicosia, Cyprus; ^7^Department of Psychiatry and Psychotherapy I, LVR-Hospital Cologne, Cologne, Germany

**Keywords:** mismatch negativity, at risk for psychosis, EEG, source analysis, risk prediction

## Abstract

**Background:**

Deficits of mismatch negativity (MMN) in patients with schizophrenia have been demonstrated many times and there is growing evidence that alterations of MMN already exist in individuals at risk for psychosis. The present study examines differences in MMN between subjects fulfilling ultra-high risk (UHR) or only basic symptoms criteria and it addresses the question, if MMN source analysis can improve prediction of transition to psychosis.

**Methods:**

The MMN to duration, frequency, and intensity deviants was recorded in 50 healthy controls and 161 individuals at risk for psychosis classified into three subgroups: only basic symptoms (*n* = 74), only ultra-high risk (*n* = 13) and persons who fulfill both risk criteria (*n* = 74). Based on a three-source model of MMN generation, we conducted an MMN source analysis and compared the amplitudes of surface electrodes and sources among the three groups.

**Results:**

Significant differences in MMN generation among the four groups were revealed at surface electrodes Cz and C4 (*p* < 0.05) and at the frontal source (*p* < 0.001) for duration deviant stimuli. The 15 subjects from the risk groups who subsequently developed a manifest psychosis had a significantly lower MMN amplitude at frontal source (*p* = 0.019) without showing significant differences at surface electrodes. Low activity at frontal MMN source increased the risk of transition to manifest disease by the factor 3.12 in UHR subjects.

**Conclusion:**

MMN activity differed significantly between subjects presenting only basic symptoms and subjects which additionally meet UHR criteria. The largest differences between groups as well as between individuals with and without transition were observed at the frontal source. The present results suggest that source analysis is more sensitive than surface electrodes in psychosis risk prediction by MMN.

## 1. Introduction

Several studies suggest a continuum of severity of psychotic symptoms ranging from subclinical psychotic symptoms (SPS) without treatment indication up to manifest schizophrenia (1–3). While SPS are common in the general population (4), schizophrenia is a rare disease with a lifetime prevalence of 0.4–0.7% (5, 6). SPS often are temporary and subtle, and only a small amount of persons with those symptoms really develop a clinically relevant psychotic disorder (7). The prodromal period of psychosis is large and subtle psychopathological changes as well as cognitive impairment can occur years before a manifest schizophrenia is diagnosed (8).

Two approaches are especially common in the field of early recognition of psychosis. Basic symptoms (BS) as subtle, subclinical disturbances in amongst others thinking and stress tolerance are an integral part of psychosis and appear through several stages of the disorder (9). While they are not specific for psychosis, a meta-analysis (10) revealed a higher conversion rate than samples established by ultra-high risk criteria. Subjects with ultra-high risk (UHR) for conversion to psychosis show attenuated psychotic symptoms (APS), brief limited intermittent psychotic symptoms (BLIPS) or trait vulnerability criteria additionally to the basic SPS and are thus also selected by clinical criteria only in current practice. Individuals with UHR have a greatly increased risk of imminent transition to a manifest psychotic disorder. However, the exact risk of transition has varied across studies (11). A recent meta-analysis shows that approximately 15 to 25% of individuals at risk for psychosis will transition to a manifest psychotic disorder within 1 to 3 years (12).

The two risk approaches complement each other. The UHR criteria were designed to detect an imminent risk for transition into a manifest schizophrenic disease. Whereas basic symptom criteria were developed with the aim of identifying the potential risk for a psychotic illness as early as possible, ideally before functional impairments occur (10). It is assumed that in some affected persons basic symptoms occur earlier in the course of the disease and that additionally attenuated psychotic symptoms (APS) and brief limited intermittent psychotic symptoms (BLIPS) occur later in the course (11). However, there are also cases with no or only mild basic symptoms which still fulfil the UHR criteria and thus have also a high risk of transition. Thus, individuals with only basic symptoms are at lower risk of imminent transition to psychosis than individuals who meet both BS and UHR criteria and those who meet UHR criteria only.

At present, clinical early recognition – as usual in general clinical diagnostic in psychiatry – is mainly based on psychopathological symptoms. Thus, UHR criteria as well as basic symptom criteria are mainly based on psychopathological symptoms. Finding biomarkers that may help to identify individuals with an increased risk of conversion to psychosis at an early stage of disease is important, because early intervention may prevent or delay the conversion to psychosis (13).

The mismatch negativity (MMN) is a component of the auditory evoked event-related potential that occurs in response to any discriminable change in an ongoing uniform acoustic stimulation, typically in the range of 100–250 ms after the stimulus (14). The MMN can be observed even in the absence of attention or in sleeping subjects, as it is generally considered to reflect the outcome of a pre-conscious change detection mechanism (15).

MMN deficiency is one of the most robust findings in schizophrenia (16). Already in the at risk state of psychosis with impairment in cognitive functions and attenuated psychotic symptoms, a MMN deficiency can be observed (17). Bodatsch et al. (18) showed that in particular the amplitude of the duration MMN is reduced in at-risk subjects, which are later converted to a manifest psychosis compared with nonconverters. Shaikh et al. (19) found that the MMN amplitude of individuals with an “at-risk mental state” was reduced compared with healthy controls. A recent large study with 580 individuals at risk for psychosis found that MMN amplitude deficits were sensitive to future psychosis conversions, particularly those not taking antipsychotic medication at baseline (20).

Several studies found an association between decreased MMN in manifest psychosis and daily functioning, social functioning and cognitive impairment (21–23). Research has suggested that duration MMN amplitude correlates with global functioning already in early stages of psychosis (24). Only few studies found an association between psychotic symptoms and changes in MMN. For instance, Donaldson et al. (25) found transdiagnostic associations between reduced duration MMN and psychotic symptoms like auditory hallucinations and disorganization. A recent study of first-episode schizophrenic patients found a correlation between duration MMN at baseline and symptom severity after 3 years, thus MMN may also be used as a predictor of remission in schizophrenia (26).

Predictive coding theories suggest that the perceptual system is a set of hierarchically organized generative models where each model provides predictions about the state of the level below. The difference between model prediction and the actual input lead to a prediction error. Event-related potentials elicited by deviant stimuli are thought to be a correlate of prediction error at an intermediate level in the hierarchy. The repetition of standard stimuli leads to suppression of prediction error and reduction of the MMN wave. When a deviating stimulus is presented a prediction error is generated again and the MMN wave emerges (27, 28). MMN can thus be thought to reflect this underlying predictive coding process. The predictive coding framework can be used to explain both MMN reduction in psychosis and development of psychotic symptoms (25, 29).

There is also evidence that in individuals at ultra-high-risk for psychosis the amplitude of the MMN induced by a frequency-deviant sound decreases with transition to psychosis (30). It can be concluded that alterations in the MMN could be useful to determine which subjects at risk are most likely to develop a psychosis and to initiate risk-adapted prevention in the clinical work. Furthermore, it has been suggested by Kim et al. (31), that in subjects at clinical high risk MMN could be used not only as predictor of transition to psychosis but also as a predictor of remission regardless of transition.

The present non-invasive electrophysiological study examines MMN in subjects at risk for psychosis fulfilling ultra-high risk (UHR) or/and basic symptoms criteria. It addresses the questions if there are differences in MMN between the different risk criteria and if MMN source analysis can improve prediction of transition to psychosis.

## 2. Methods

### 2.1. Subjects and assessment

Individuals at risk for psychosis were recruited as part of the multimodal ZInEP (Zurich Program for Sustainable Development of Mental Health Services) early recognition study (32). Subjects were recruited by a study website, advertisements in newspapers and flyers or a clinical therapist assigned the subjects to the study center. At study baseline amongst others psychopathology and neuropsychology were measured and EEG was recorded (33, 34). All interviews, cognitive testing and EEG measurement were administered by experienced and extensively trained psychologists and psychiatrists.

Inclusion criteria for the present study were individuals aged 13–35 years, sufficient German speaking ability, fulfilling at least one of the following psychosis risk criteria: (1) basic symptoms (BS), with at least one cognitive-perceptive (COPER) basic symptom or at least two cognitive disturbances (COGDIS) basic symptoms, assessed by the adult (35) or children-youth (36) version of the Schizophrenia Proneness Interview (SPI-A/SPI-CY), (2) ultra-high-risk status for psychosis (UHR) was rated by the Structured Interview for Prodromal Syndromes - SIPS (37), with at least one attenuated psychotic symptom, or at least one brief limited intermittent psychotic symptom, or a positive state–trait criterion (reduction in global assessment of functioning of >30% in the past year, plus either schizotypal personality disorder or first degree relative with psychosis).

The two groups were created to distinguish between individuals with a general risk (BS) and individuals with imminent risk (UHR) of transition to manifest schizophrenia (11, 38). All subjects at risk were followed up over 3 years as part of the ZInEP early recognition study (32, 39) to detect transitions in a manifest psychotic disorder. Transition to psychosis was defined according to ICD-10 criteria for schizophrenia. The diagnosis schizophrenia was made if at least one so-called Schneider’s first rank symptom or at least two other symptoms of schizophrenia were present for most of the time during an episode lasting for at least 1 month.

Exclusion criteria were: estimated premorbid IQ < 80, meeting DSM-IV criteria for current substance dependence, any psychotic disorder confirmed by research diagnostic interviews, and/or any medical condition known to affect the brain.

Healthy controls matching age and gender were included in the study. A Mini-International Neuropsychiatric Interview (40) was used to assure the absence of any mental illness in control subjects.

The study was approved by the ethics committee of the canton Zurich and carried out in accordance with the Declaration of Helsinki. All participants gave their written informed consent after receiving a detailed description of the study and in case of minors the written informed consent was obtained from their parents too.

### 2.2. EEG recording

Subjects were tested in a quiet laboratory, sitting in a comfortable chair. EEG data were recorded using a BrainAmp amplifier and Brain Vision Recorder Software. Thirty-two Electrodes were applied to the scalp by well-trained professionals and held in position by a nylon cap (BrainCap MR32 standard; EASYCAP, Herrsching-Breitbrunn, Germany). EEG channels were referenced to FCz, scalp electrode impedance was kept below 10 k. An EOG electrode was positioned below the right eye and ground was positioned at AFz. The sampling rate was 500 Hz. A band-pass filter of 0.1 to 100.0 Hz (12 dB/ octave rolloff each) was applied to collect the data. 2,400 acoustic stimuli were presented binaurally by headphones and Presentation software (Neurobehavioral Systems, Inc., San Pablo, CA, United States). During recording, participants were instructed to relax and watch a soundless movie clip of “Mr. Bean” presented on an easily visible screen to distract attention away from the acoustic stimuli. The acoustic stimuli included 1896 standard (1,000 Hz, 100 ms, 80 dB; 79% of total stimuli), 168 duration-deviant (1,000 Hz, 50 ms, 80 dB; 7% of total stimuli), 168 frequency-deviant (1,200 Hz, 100 ms, 80 dB; 7% of total stimuli), and 168 intensity-deviant tones (1,000 Hz, 100 ms, 70 dB; 7% of total stimuli), which were applied in a pseudo-random sequence without recurring order as one continuous block. There were at least two standard stimuli between each deviant stimulus and the stimulus onset asynchrony was 500 ms. The participant was observed closely during the 20 min of EEG-recording.

### 2.3. Data preprocessing and analysis

The recorded EEG files were edited using Brain Electrical Source Analysis (BESA) software, version 5.3. The EEGs were re-referenced to an average reference. Before averaging the EEGs, a filter with the low cut-off of 1 Hz and a high cut-off of 20 Hz (both 12 dB/ octave) was applied. Then each EEG file was divided into 500 ms epochs including a 100 ms pre-stimulus baseline interval and blinking artefacts were eliminated. All trials with amplitudes exceeding 120 V were discarded, all EEG files were visually examined and if the horizontal or vertical EOG channels detected eye movement, the corresponding EEG epoch was declined. Subject providing less than 60% accepted trials were excluded from the study. The included trials were averaged individually for each subject and each condition (standard and deviant in duration, frequency or intensity). Afterwards individual standard and MMN average waveforms were calculated for every subject and every condition. The standard average waveform was subtracted from the particular deviant waveforms, namely duration, frequency or intensity, resulting in the respective MMN waveforms. The MMN waveforms at six centrally positioned surface electrodes (Fz, F3, F4, Cz, C3, and C4) (41) were examined and the peak MMN amplitude and latency were determined. Peak amplitude was detected within a window of 150–250 ms post-stimulus. This was performed for each group of subjects and each condition separately.

### 2.4. Source analysis

For the Source Analysis we used the BESA spatiotemporal source analysis tool in accordance with the BESA tutorial by Hoechstetter et al. (42). We assumed a source model with two symmetric regional sources temporal in the auditory cortex, based on knowledge that MMN is generated in the primary auditory cortex, and a third regional source located in the frontal cortex, as it is suitable for MMN (43, 44), assuming a contribution in generating MMN made by the right frontal cortex. We used MRI image CLARA (“Classical LORETA Analysis Recursively Applied”), an iterative application of the LORETA (“Low-resolution electromagnetic tomography”) algorithm, in which the source space is implicitly reduced in each iteration. Using the grand average of all subjects a source model was created for each condition. Then the event related potentials of each subject were used together with the source model acquired before out of the grand averages to asses individual MMN source activity for each participant and each condition so that potential differences among the study groups could be evaluated.

### 2.5. Statistical analysis

Demographic and clinical characteristics were analyzed using Chi-square statistics for categorical variables and one-way analysis of variance (ANOVA) for continuous variables. Distribution of MMN surface activity (Fz, F3, F4, Cz, C3, and C4) and MMN source activity (RS1, RS2, and RS3) were compared across groups using one-way ANOVAs. Pairwise group comparisons were performed using Bonferroni post-hoc comparisons for continuous data. Unadjusted and adjusted (for demographic and clinical variables) logistic regression models were conducted for subjects meeting the UHR criteria to estimate transition probability according to MMN source activity in the duration condition. For regression analyses measures of MMN source activity were inverse coded and, as well as other continuous variables, centered to sample mean (*z*-transformed).

All statistical analyses were performed using STATA/SE 16.0 (StataCorp LP, TX, United States).

## 3. Results

### 3.1. Sample

One-hundred sixty-one individuals at risk for psychosis could be included in the study. Of these, *n* = 74 subjects fulfilled only the basic symptom (BS) criteria and *n* = 13 were classified as only ultra-high risk (UHR), while *n* = 74 met both UHR and BS criteria. The control group consisted of 50 healthy controls matched by age and gender (see Table 1). The UHR only and the combined UHR&BS group were significant younger and reported more positive symptoms on the SIPS than the only BS group. The combined UHR&BS group had more SIPS negative symptoms and lower functioning than the BS group.

**Table 1:** Demographic and clinical characteristics of the study sample.

	Controls		At-risk			Group comparisons	
	CON	All at-risk (BS, UHR & BS, UHR)	BS	UHR & BS	UHR	CON vs. at-risk value of *p*	Across subgroups overall value of *p*
*n*	50	161	74	74	13		
Gender male	27 (54.0%)	97 (60.25%)	46 (62.16%)	41 (55.41%)	10 (76.92%)	*p* = 0.433	*p* = 0.402
Age	21.00 ± 5.55	20.70 ± 5.65	23.11 ± 5.69	18.91 ± 4.86	17.15 ± 3.98	*p* = 0.741	**p < 0.001** BS > UHR&BS***; UHR*
SIPS positive	–	7.94 ± 4.62	4.58 ± 3.29	10.86 ± 3.68	10.44 ± 2.68	–	**p < 0.001**^a^ UHR&BS; UHR > BS***
SIPS negative	–	11.94 ± 6.13	10.46 ± 5.95	13.32 ± 6.01	12.46 ± 6.36	–	***p* = 0.016** ^a^ UHR&BS > BS*
GAF	–	55.61 ± 13.67	58.92 ± 14.75	52.01 ± 12.19	57.67 ± 10.51	–	***p* = 0.008** ^a^ BS > UHR&BS***
CPZ-equivalent	–	26.29 ± 142.9	10.03 ± 37.81	47.18 ± 206.12	0 ± 0	–	*p* = 0.227 ^a^
Transition F20 (n/%)	–	15 (9.32%)	3 (4.05%)	10 (13.51%)	2 (15.38%)	–	*p* = 0.104 ^a^

CON, control group; BS, only basic symptoms criteria; UHR&BS, fulfilling basic symptoms criteria and ultra-high-risk criteria; UHR, only ultra-high-risk criteria; SIPS, structured interview for prodromal syndromes; GAF, global assessment of functioning; CPZ-equivalent, chlorpromazine equivalent; Transition F20, transition into manifest schizophrenia.

a (comparisons were done without CON), *(*p* < 0.05) and ***(*p* < 0.001).

### 3.2. MMN surface amplitudes

**Figure 1:**
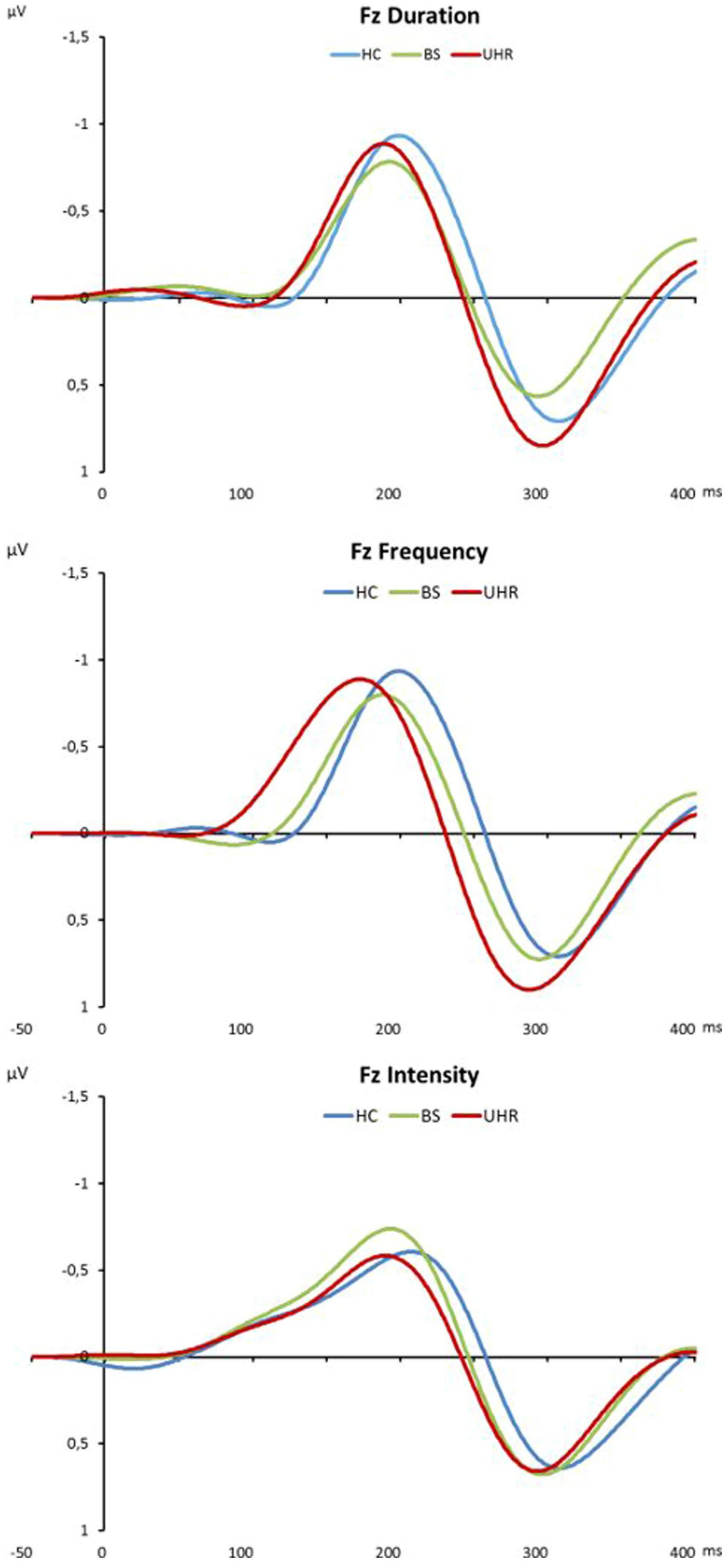
Grand average MMN surface waveforms for duration, frequency and intensity deviants in microvolts at electrode Fz. HC, healthy controls; BS, basic symptom group; UHR, ultra-high-risk group (UHR&BS + only UHR).

Grand average MMN surface waveforms for duration, frequency and intensity deviants are displayed in Figure 1. Mean peak amplitudes (±standard deviation) for the duration deviant condition are presented for the six examined electrodes in Table 2. No significant differences in MMN surface amplitudes were found when comparing the whole risk group with the control group. In comparison across all subgroups, significant amplitude differences were found at electrodes Cz and C4. Bonferroni-corrected pairwise post-hoc comparisons revealed significantly lower amplitude in the BS group compared with the UHR&BS group (significant for electrode Cz). No significant group differences at surface electrodes were found for the two other deviant conditions intensity and frequency.

**Table 2:** MMN peak amplitudes (μV) and source activity (nAm) ± standard deviation.

	Controls		At-risk (sub groups)			Group comparison	
	CON	All at risk (BS, UHR, UHR & BS)	BS	UHR & BS	UHR	CON vs. all	Across subgroups (CON, BS, UHR, UHR & BS)
Surface electrodes (μV mean ± SD)						Value of *p*	overall value of *p*
Fz	−1.32 ± 0.56	−1.31 ± 0.55	−1.19 ± 0.51	−1.41 ± 0.56	−1.40 ± 0.64	*p* = 0.920	*p* = 0.109
F3	−1.15 ± 0.53	−1.17 ± 0.54	−1.09 ± 0.49	−1.25 ± 0.58	−1.20 ± 0.50	*p* = 0.816	*p* = 0.338
F4	−1.29 ± 0.63	−1.36 ± 0.55	−1.25 ± 0.51	−1.44 ± 0.55	−1.44 ± 0.76	*p* = 0.491	*p* = 0.187
Cz	−1.16 ± 0.51	−1.05 ± 0.47	−0.94 ± 0.43	−1.15 ± 0.48	−1.15 ± 0.54	*p* = 0.173	***p* = 0.022** BS > UHR & BS*
C3	−1.03 ± 0.40	−0.97 ± 0.50	−0.87 ± 0.43	−1.08 ± 0.55	−0.90 ± 0.52	*p* = 0.441	*p* = 0.052
C4	−1.12 ± 0.56	−1.00 ± 0.48	−0.89 ± 0.48	−1.10 ± 0.48	−1.05 ± 0.37	*p* = 0.130	***p* = 0.028** no sig. *Post hoc*
Sources (nAm mean ± SD)							
Left temporal (RS1)	14.71 ± 8.51	16.11 ± 7.45	14.86 ± 7.48	16.96 ± 7.13	18.33 ± 8.47	*p* = 0.266	*p* = 0.159
Right temp. (RS2)	13.08 ± 5.54	14.69 ± 7.35	13.66 ± 6.38	15.74 ± 7.79	14.56 ± 9.51	*p* = 0.156	*p* = 0.150
Frontal (RS3)	13.95 ± 7.17	8.34 ± 4.42	7.72 ± 4.16	8.80 ± 4.42	9.23 ± 5.65	**p < 0.001**	**p < 0.001** CON > BS*** CON > BS & UHR*** CON > UHR*

CON, control group; BS, only basic symptoms criteria; UHR&BS, fulfilling basic symptoms criteria and ultra-high-risk criteria; UHR, only ultra-high-risk criteria; RS, regional source; *(*p* < 0.05) and ***(*p* < 0.001).

### 3.3. MMN source activity

The BESA source localization revealed three regional sources (RS): one in the left superior temporal lobe (RS1), one in the right superior temporal lobe (RS2), and a third in the anterior cingulate gyrus (RS3). Transferred to the Talairach space, the first two sources were based in the primary auditory cortices (Brodmann 41) on the left and right transverse temporal gyri and the third source in the anterior cingulate area (Brodmann 24).

Comparing MMN Source activity between individuals at risk and healthy controls group differences were found only in the duration condition for the activity of the frontal regional source (RS3; Table 2). Source activity was significantly lower in individuals at risk compared to controls (RS3-posthoc: CON > BS, *p* < 0.001; CON > UHR&BS, *p* < 0.001; CON > UHR, *p* = 0.024).

Correlation analyses between demographic variables, functioning and MMN source activity in the duration condition found gender to be linked to lower activity in the right temporal source (RS2), while age was linked to lower activity in the left temporal source (RS1; Table 3). Higher activity in the left temporal source (RS1) was positively associated to activities in the two other sources (RS2 & RS3). Baseline global functioning (GAF) was not bi-variately related to any other study variable.

**Table 3:** Correlation analysis of the three MMN sources activity in the duration condition with age, sex, and global functional level.

	1. Sex	2. Age	3. RS1 (duration)	RS2 (duration)	RS3 (duration)	GAF Baseline
Sex	–					
Age	0.086	–				
RS1 duration	−0.085	−0.169*	–			
RS2 duration	−0.141*	−0.108	0.407***	–		
RS3 duration	−0.130	−0.073	0.236***	0.088	–	
GAF baseline	0.001	0.100	−0.027	0.003	0.000	–

RS1, left temporal source; RS2, right temporal source; RS3, frontal source; GAF, global assessment of functioning (only available for subjects at risk). *p < 0.05 and ***p < 0.001.

### 3.4. Transition versus no transition

**Figure 2:**
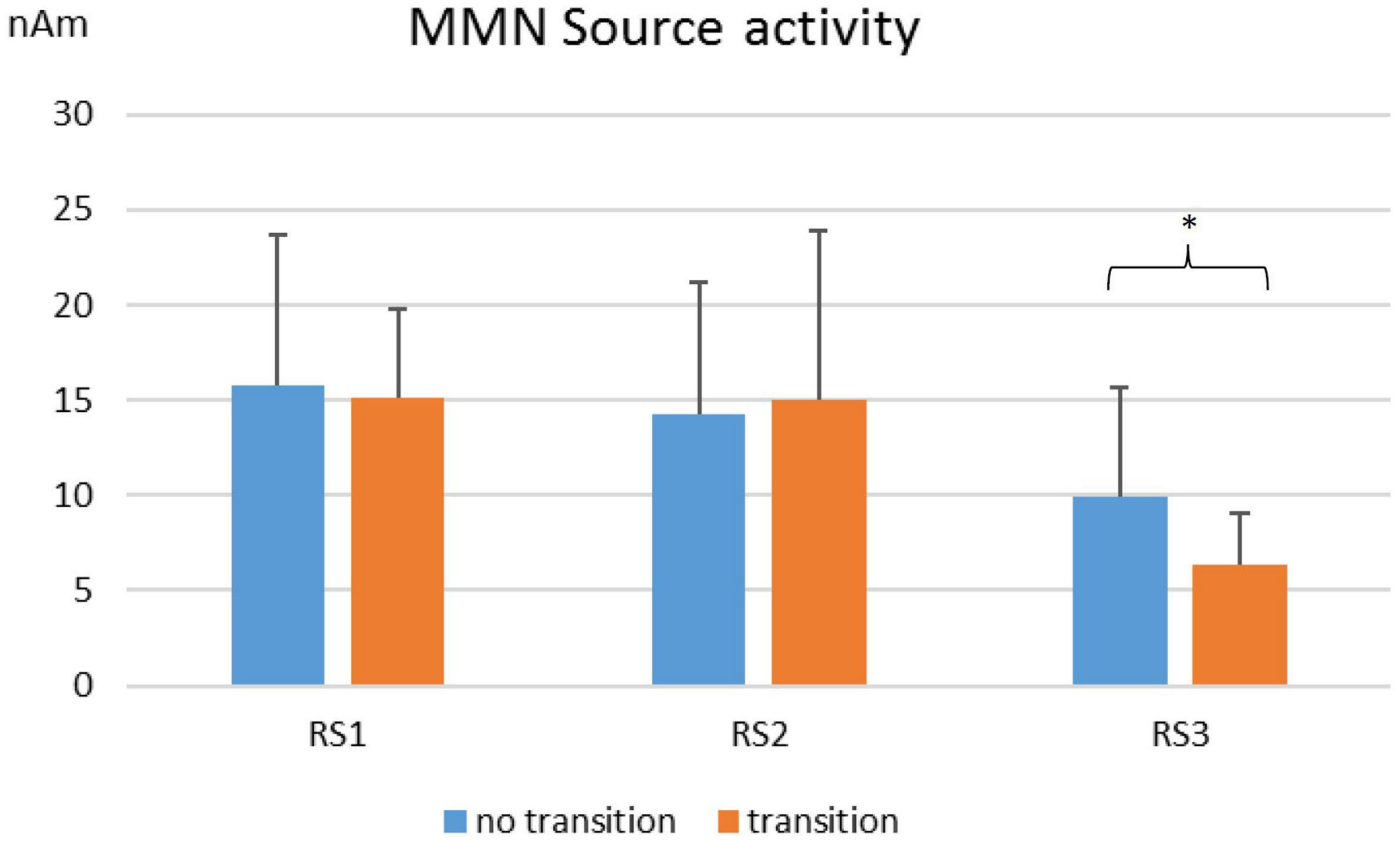
MMN source activity in nAm for duration deviants. Comparison of individuals with (*n* = 15) and without transition (*n* = 146) to manifest schizophrenia. RS1, left regional source; RS2, right regional source; RS3, frontal regional source. *p < 0.05.

The fifteen individuals with transition to manifest schizophrenia did not differ from subjects at risk without transition in MMN activity at surface electrodes. With respect to MMN source activity, a significant difference was found only at the frontal source (RS3) in the duration condition with a lower MMN source activity in subjects with transition (*F* = 5.601; *p* = 0.019) (Figure 2).

Table 4 shows the results from logistic regression models estimating the transition probability in subjects fulfilling the UHR criteria according to MMN source activity in the duration condition, sex, age, psychopathology and global functioning. Unadjusted models revealed lower frontal source (RS3) activity to increase the likelihood for F20 transition by the factor 3.12, while no other predictor was linked to F20 transition. Effect for RS3 increased after adjusting for all other variables, while age as well as SIPS positive symptoms were also found to be linked to transition in the adjusted model.

**Table 4:** Results of logistic regression models estimating transition probability in UHR individuals.

	No Transition (*n* = 75)	Transition F20 (*n* = 12)	Unadj. OR (95%CI)	Adj. OR (95%CI)
Sex Male (*n*/%)	43 (57.3%)	8 (66.7%)	1.49 (0.41–5.38)	1.35 (0.25–7.34)
Age	18.37 ± 4.52	20.33 ± 6.00	1.55 (0.80–2.99)	2.97 (1.18–7.45)*
RS1 duration (invers coded)	−16.62 ± 7.55	−13.34 ± 4.92	1.80 (0.81–4.00)	1.45 (0.43–4.87)
RS2 duration (invers coded)	−14.38 ± 7.87	−15.66 ± 9.20	0.87 (0.52–1.46)	0.53 (0.25–1.13)
RS3 duration (invers coded)	−8.29 ± 4.68	−5.21 ± 2.89	3.12 (1.08–9.07)*	5.34 (1.16–24.60)*
GAF baseline	52.69 ± 11.93	53.64 ± 13.57	1.09 (0.53–2.26)	1.89 (0.77–4.64)
SIPS positive	10.57 ± 3.48	12.23 ± 3.69	1.79 (0.83–3.87)	3.21 (1.09–9.41)*
SIPS negative	12.88 ± 5.81	15.17 ± 7.25	1.49 (0.78–2.85)	0.90 (0.38–2.10)

OR, odds ratio; 95%CI, 95% confidence interval; GAF, general assessment of functioning; RS, regional source; SIPS, structured interview for prodromal syndromes; *p < 0.05.

## 4. Discussion

In recent years, several studies have shown the predictive value of MMN for assessing the risk of transition in individuals at risk for psychosis (18–20, 45–49). The present study extends the existing knowledge by examining differences in MMN between different risk groups, subjects fulfilling only basic symptoms, subjects who meet both BS and UHR criteria and subjects only at ultra-high-risk for psychosis. In addition, a source analysis of MMN was performed to determine whether evaluation of source activity can improve risk assessment. Three different deviant stimuli (duration, frequency, intensity) in a traditional constant standard MMN paradigm involving the same high probability standard stimulus throughout the whole sequence were used in the study. Significant differences could be detected only for duration deviants.

Subjects from the only basic symptoms group had a significantly lower MMN amplitude compared to the group fulfilling both UHR & BS in the analysis of the surface electrodes. No significant differences were found between persons at risk for psychosis and controls at surface electrodes. However, when the underlying source activity of MMN was examined, significant differences were found at the frontal source between controls and all three risk groups.

Dipole modeling studies as well as fMRI and PET investigations have shown that in addition to both temporal generators, a frontal generator is also involved in the development of MMN (50–55). Some authors assign the frontal components of MMN the role of directing attention on detection of changes in sensory processing areas (56, 57). Within the framework of hierarchical predictive coding theory, it is assumed that the MMN reflects an error signal. This error signal occurs when a sensory input does not match the prediction for that input (58). Frontal mechanisms are thought to underlie the coding of the predicted representation, which then acts on sensory processing regions (59).

The results of the present study suggest that particularly the frontal components of MMN are disturbed in the risk state for psychosis. All three risk groups showed significantly lower MMN activity at frontal source compared to healthy controls. The fact that this change was already detectable in the only BS group indicates that changes in MMN occur already early in the course of the disease. Disturbances of frontal brain functions belong to the typical characteristics of the schizophrenic disease (60, 61). Consistent with this is the finding that individuals with transition to manifest schizophrenia showed the strongest alterations at the frontal MMN source already in the risk state.

During the observation period of 3 years after MMN examination, 15 participants developed a manifest schizophrenic disorder. Individuals with transition already differed at the time of study inclusion by significantly lower activation of the frontal MMN source from individuals in the risk state of psychosis who did not develop manifest schizophrenia during the observation period. Due to the limited follow-up period of 3 years, only a very small number of transitions (*n* = 3) could be observed in the only BS group. The analysis of transition probability was therefore limited to those individuals who met UHR criteria. The logistic regression model showed that low activity at the frontal MMN source more than tripled the probability of transition in UHR subjects. Transferred to the average transition risk of 15 to 25% within 3 years (12), this could mean that individuals who are at risk for psychosis and additionally have low activity at the frontal MMN source might have a risk of about 45 up to over 75% of transition into manifest psychosis.

A limitation of the study is that localization of sources by EEG is imprecise. This may account for the different localization of frontal MMN source in various studies [e.g., middle frontal gyrus, left, right, or bilateral inferior frontal gyrus and anterior cingulum (50–52, 62, 63)]. However, this variability in the location of the frontal source could also stem from variations in the degree of attentional focus on the stimuli (59). A further limitation arises from the circumstance that some of the individuals in the at-risk state were already receiving antipsychotic medication. However, the average chlorpromazine equivalent was relatively low at 26.3 mg per day. Another limitation is the relatively low transition rate of 9.3 percent, which is still in line with other early recognition studies (12, 39).

The present study found significant changes only for duration deviant stimuli. This is in line with other studies which reported stronger MMN changes in individuals at risk for psychosis to duration deviant stimuli compared to frequency deviant stimuli (18, 64). A possible explanation could be that processing of duration changes requires more complex brain functions than processing of frequency changes, and more complex processes can already be affected by discrete brain dysfunctions as they are present in risk states for psychosis.

## 5. Conclusion

Consistent with the existing literature, the present study was able to confirm MMN alterations in individuals at risk for psychosis. Through analysis of the underlying source activity, these changes could be attributed primarily to the frontal MMN source. Alterations in the frontal components of MMN appear to be particularly relevant for predicting a transition to manifest schizophrenic disorder. Even if MMN is not suitable as a sole biomarker of psychosis, it may contribute additional information about the risk of transition in individuals fulfilling ultra-high risk (UHR) for psychosis.

## Data availability statement

The datasets presented in this article are not readily available because they are not publicly available. Requests to access the datasets should be directed to the the corresponding author karsten.heekeren@uzh.ch.

## Ethics statement

The study was reviewed and approved by the Ethics committee of the canton of Zurich (KEK-ZH-Nr. E-63/2009). Written informed consent was obtained from all participants and participants’ parents/legal guardians for their participation in this study.

## Author contributions

TA, AT, SW, WR, WK, and KH designed the study and wrote the protocol. TA and FH collected the data. TA, MM, and KH analyzed the data. TA drafted the manuscript. TA, MM, AT, FH, ES, SW, WR, WK, and KH discussed the results and reviewed the manuscript, making critical revisions. All authors contributed to the article and approved the submitted version.

## Funding

The Zurich Program for Sustainable Development of Mental Health Services (ZInEP) was supported by a private donation. The donor had no further role in the experimental design, collection, analysis, interpretation of data, writing, and submitting this paper for publication.

## Conflict of interest

The authors declare that the research was conducted in the absence of any commercial or financial relationships that could be construed as a potential conflict of interest.

## Publisher’s note

All claims expressed in this article are solely those of the authors and do not necessarily represent those of their affiliated organizations, or those of the publisher, the editors and the reviewers. Any product that may be evaluated in this article, or claim that may be made by its manufacturer, is not guaranteed or endorsed by the publisher.
